# Rice stripe virus counters reduced fecundity in its insect vector by modifying insect physiology, primary endosymbionts and feeding behavior

**DOI:** 10.1038/srep12527

**Published:** 2015-07-27

**Authors:** Guijun Wan, Shoulin Jiang, Wenjing Wang, Guoqing Li, Xiaorong Tao, Weidong Pan, Gregory A. Sword, Fajun Chen

**Affiliations:** 1 Department of Entomology, College of Plant Protection, Nanjing Agricultural University, Nanjing 210095, China; 2 Department of Plant Pathology, College of Plant Protection, Nanjing Agricultural University, Nanjing 210095, China; 3 Beijing Key Laboratory of Bioelectromagetics, Institute of Electrical Engineering, Chinese Academy of Sciences, Beijing 100190, China; 4 Department of Entomology, Texas A&M University, College Station, TX 77843, USA

## Abstract

Virus-vector relationships can be complex and diverse as a result of long-term coevolution. Understanding these interactions is crucial for disease and vector management. Rice stripe virus (RSV) is known to be transovarially transmitted within its vector, *Laodelphax striatellus,* and causes serious rice stripe disease. In RSV-infected *L. striatellus*, we found contrasting changes in vector fecundity, physiology, primary endosymbionts (i.e. yeast-like symbionts, YLS) and feeding behavior that can interact to affect the spread of RSV. RSV-infected *L. striatellus* exhibited a significant decrease in fecundity that could lead a reduction of viruliferous individuals in populations. As a potential response to this loss, RSV infection also significantly shortened nymphal stage duration, which can strengthen RSV vertical circulation in *L. striatellus* populations and promote RSV spreading by adult migration and dispersal. Down-regulated *JHAMT* and up-regulated *CYP307A1* in the juvenile hormone and ecdysteroid pathways, respectively, were linked to accelerated development. RSV-infected adults were also found to have higher body weight in conjunction with increased YLS abundance. Furthermore, prolonged host plant phloem exposure to salivation by RSV-infected adults should further enhance RSV horizontal transmission. Our study highlights potential strategies of RSV in enhancing its transmission, and provides new insights into the complexity of virus-vector interactions.

Approximately 80% of plant viruses are known to spread through insect vectors^1^. The physiology, behavior and even endosymbionts of insect vectors can have profound ecological and evolutionary implications for the transmission of a plant virus to a new host. The migratory small brown planthopper, *Laodelphax striatellus*, is an insect with a high dispersal ability that has been an increasing threat to rice yield since the 1960s and acts as the main insect vector of rice stripe virus (RSV)^2^,^3^,^4^. As a member of the genus *Tenuivirus*, RSV is persistent across generations and transovarially transmitted in a circulative-propagative manner in *L. striatellus*^5^. Its transmission to rice has caused severe reductions in yield over the last few decades in China. It was reported that RSV ribonucleoproteins are distributed throughout the ovum, lumen and epithelial cells of midgut and even muscles^6^, and consistent with its transovarial transmission, RSV particles are also found in follicular cells of ovarioles of *L. striatellus*^6^. Repeated transovarial passage of RSV was demonstrated to last for 40 generations in planthoppers, and *L. striatellus* females were more efficient in RSV transmission than males^7^. Nevertheless, many fundamental questions about RSV-*L. striatellus* interactions remain to be addressed including the nature and consequences (if any) of other phenotypic effects of RSV infection on *L. striatellus.*

Recently *vitellogenin* (*Vg*) was demonstrated to play a critical role in assisting the virus to enter nurse cells in the germarium in *L. striatellus* via *Vg*-mediated endocytosis^8^. After incorporating into oocytes, *Vg* is stored in a crystalline form of *Vitellin (Vn)* and reserved as a food source for the future embryo^9^. Thus, *Vg* is very important for the propagation and transmission of RSV due to the direct relationship between *Vg* production and the vector’s fecundity and abundance at the population level. Importantly, most hemipteran insects use only juvenile hormone (JH) to regulate *Vg* gene transcription^10^. JH and the main ecdysteroid, 20-hydroxy-ecdysone (20E), are highly versatile insect hormones that coordinate development, growth and reproduction of insects^11^. It has also been reported that 20E and JH play critical roles in innate immunity^12^,^13^. Therefore, in this study we use expression patterns of three genes, *JHAMT* in the juvenile hormone pathway, *CYP307A1* in the ecdysteroid pathway, and *Vg* to assess the potential immune response to RSV infection as well as the effects of RSV infection on the development and fecundity of *L. striatellus* at the transcriptional level.

The potential for interactions between RSV and yeast-like symbionts (YLS) in the *L. striatellus* system remains an open question. As primary endosymbionts that are critical to *L. striatellus* biology, YLS are also transovarially transmitted in *L. striatellus*^14^,^15^,^16^. YLS provide their host with nutritional benefits, and their absence can have adverse impacts on planthopper performance^17^,^18^,^19^. For instance, *L. striatellus* can obtain sterols from those synthesized by YLS^18^,^19^,^20^, whereas insects that do not harbor YLS have to ingest exogenous sterols from food^21^ that are necessary for cellular membrane structures and as precursors for ecdysteroids^20^,^22^. As YLS and RSV are both located in the same tissues^6^ and transovarially transmitted in *L. striatellus*^8^,^15^, we tested for potential interactions between them during the life cycle of *L. striatellus*.

Evidence currently indicates that the relationships between a virus and vector can be either mutualistic^23^,^24^ or antagonistic^25^,^26^, and the virus may affect the vector’s physiology and behavior to influence its likelihood of transmission^27^,^28^,^29^,^30^,^31^. Nevertheless, the lack of comprehensive and integrated consideration of vector physiology and behavior after virus infection has limited our understanding of the evolutionary significance and mechanisms underlying their interactions. In this study, we systemically examined the changes in physiology (including development, body weight and fecundity), primary endosymbionts (i.e. YLS) and feeding behavior of RSV-infected *L. striatellus* to more comprehensively understand the interactions in the RSV-*L. striatellus*-YLS system.

## Results

### Effects of RSV infection on *L. striatellus* egg hatching and nymphal stage

**Figure 1:**
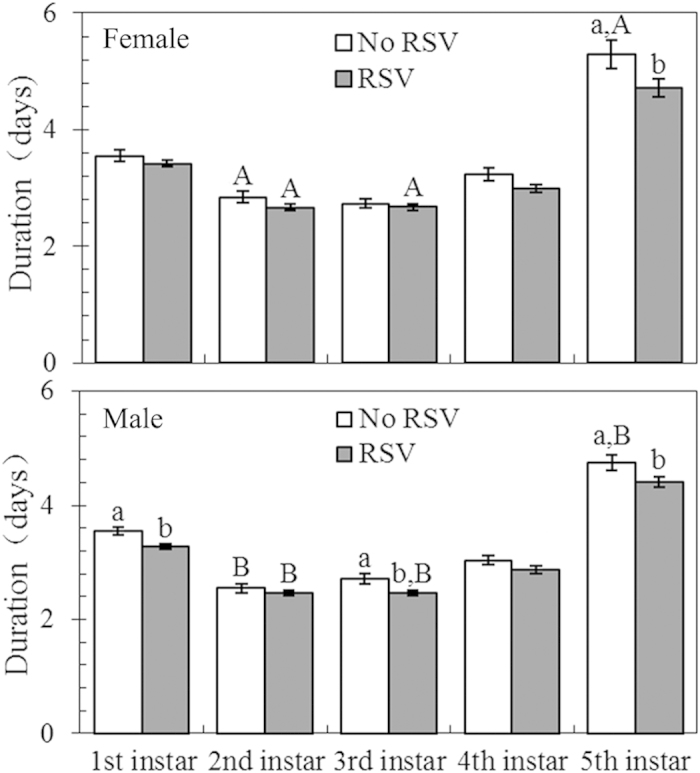
Durations of the 1st-5th instar nymphs of females and males of *L. striatellus* with (RSV) and without RSV (No RSV) infection. n = 184 and 181 for the infected females and males, respectively; n = 156 and 166 for the uninfected females and males, respectively. Only significant differences are marked with letters. Different lowercase and uppercase letters show significant differences between the infected and uninfected *L. striatellus* for females or males, and between females and males for the infected or uninfected *L. striatellus* by LSD test at *P* < 0.05.

There was no significant difference in hatching period between the viruliferous population and the non-viruliferous strains (means: 8.20 vs. 8.35; *P* = 0.34; see Supplementary Table S2 and Supplementary Fig. S2 online). However, RSV infection significantly shortened the duration of the 1st (*P* = 0.001), 3rd (*P* = 0.03), 4th (*P* = 0.01), 5th (*P* = 0.005) and total nymphal stages (*P* < 0.001). Significant effects of sex on the duration of the 2nd (*P* < 0.001), 3rd (*P* = 0.047), 5th (*P* = 0.03) and total nymphal stages (*P* < 0.001) were also found. RSV infection significantly reduced the 5th instar and total nymphal stage development time by 10.78% and 6.69%, respectively, for females, and 7.17% and 6.69% for males (all differences *P* < 0.05; Table 1, Fig. 1). Moreover, for males, the 1st and 3rd instar durations were significantly shortened by 7.87% and 9.23%, respectively (all differences *P* < 0.05; Fig. 1). Among RSV-infected 3rd instar nymphs, the male duration was significantly shorter than the female duration (mean difference: -7.87%; *P* < 0.05; Fig. 1), while no significant difference was found between the male and female durations in the absence of RSV infection (*P* > 0.05; Fig. 1). Among the 5th instar nymphs, the only significant difference was a shorter developmental period for males versus females in the absence of RSV infection (mean difference: -10.40%; *P* < 0.05; Fig. 1).

### Dynamic expression levels of genes in ecdysteroid and juvenile hormone (JH) pathways

**Figure 2:**
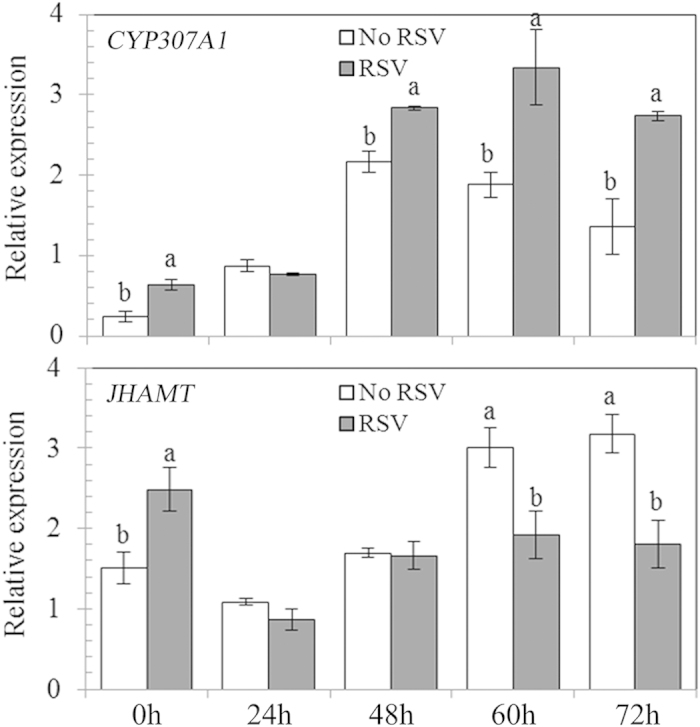
The temporal dynamics of gene expression levels of *CYP307A1* and *JHAMT* at 0 h, 24 h, 48 h, 60 h and 72 h after molting in the 5th instar nymphs of *L. striatellus,* with (RSV) and without RSV (No RSV) infection. Thirty individual 5th instar nymphs were randomly mixed as one sample for each sampling time with three repeats. Only significant differences are marked with letters. Different lowercase showed significant differences between the infected and uninfected *L. striatellus* with RSV by LSD test at *P* < 0.05.

Given that there were significant differences in the duration of the 5th instar between *L. striatellus* with and without RSV infection for both females and males, we examined the expression of two key genes in the KEGG insect hormone biosynthesis pathway, *CYP307A1* in the ecdysteroid pathway and *JHAMT* in the JH pathway, across this developmental stage. We measured expression levels at 0 h, 24 h, 48 h, 60 h and 72 h after molting into the 5th instar. For both *CYP307A1* and *JHAMT*, we found significant main effects of RSV infection (*CYP307A1*: *P* < 0.001; *JHAMT*: *P* = 0.02), sampling time (*P* < 0.001; *P* < 0.001) and their interactions (*P* = 0.004; *P* < 0.001) on gene expression levels (see Supplementary Table S3 online). Figure 2 shows that *CYP307A1* expression levels were significantly higher in nymphs with RSV infection at 0 h (mean difference: +157.15%), 48 h (mean difference: +31.26%), 60 h (mean difference: +77.78%) and 72 h (mean difference: +101.98%) after molting (all differences *P* < 0.05; Fig. 2). Differences in *CYP307A1* expression levels between 5th instar nymphs with and without RSV infection were not significant at 24 h (mean relative expression: 0.77 vs. 0.87) (*P* > 0.05; Fig. 2). *JHAMT* expression levels were significantly higher in nymphs with RSV infection at 0 h immediately after molting (mean difference: +65.04%), but significantly lower at 60 h (mean difference: –36.10%) and 72 h (mean difference: –43.35%) after molting (all differences *P* < 0.05; Fig. 2). Differences in *JHAMT* expression levels between 5th instar nymphs with and without RSV infection were not significant at 24 h (mean relative expression: 0.86 vs. 1.08) and 48 h (mean relative expression: 1.66 vs. 1.70) (both comparisons *P* > 0.05; Fig. 2).

### Effects of RSV infection on the adult weight of *L. striatellus*

Significant effects of RSV infection (*P* = 0.001) and sex (*P* < 0.001) on the body weight of newly emerged adult were found, but no significant interaction between RSV infection and sex was observed (*P* = 0.63) (see Supplementary Table S2 online). Compared with non-viruliferous individuals, RSV infection significantly increased body weight of the newly emerged female adults (mean difference: +6.82%; *P* < 0.05; Table 1). Newly emerged RSV-infected male adults were also heavier than non-RSV males (means: 0.689 mg vs. 0.677 mg), but the difference was not significant (*P* > 0.05; Table 1). Overall, the body weight of newly emerged female adults was significantly greater than that of newly emerged male adults both with (mean difference: +18.29%) and without (mean difference: +12.70%) RSV infection (both comparisons *P* < 0.05; Table 1).

### Effects of RSV infection on *L. striatellus* female fecundity and *Vg* gene expression

**Figure 3:**
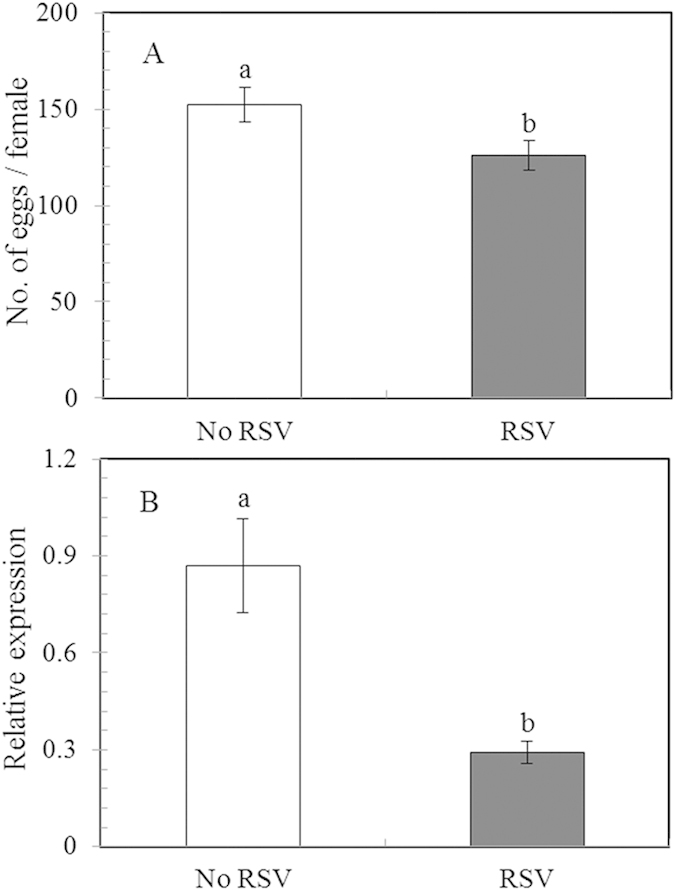
The number of eggs laid per female adults (A) and the relative transcript levels of *Vg* in newly emerged female adults (B) of *L. striatellus* with (RSV) and without RSV (No RSV) infection. n = 15 for both the infected and uninfected female adults in panel A; Ten newly emerged female adults were randomly mixed as one sample with nine repeats for each treatment in panel B. Only significant differences are marked with letters. Different lowercase letters showed significant differences between the *L. striatellus* with RSV infection and those without RSV infection by LSD test at *P* < 0.05.

RSV infection significantly affected the number of eggs laid per female (*P* = 0.007; see Supplementary Table S2 online) as well as the gene expression level of *Vg* in newly emerged adult females (*P* = 0.04; see Supplementary Table S3 online). The mean number of eggs laid per female was significantly reduced by 15.05% in RSV-infected individuals compared to those without RSV infection (*P* < 0.05; Fig. 3A). The level of *Vg* gene expression in newly emerged female adults with RSV infection was significantly lower than that of *L. striatellus* females without RSV infection (mean difference: -66.32%; *P* < 0.05; Fig. 3B).

### Effects of RSV infection on the abundance of YLS in *L. striatellus* adults

**Figure 4:**
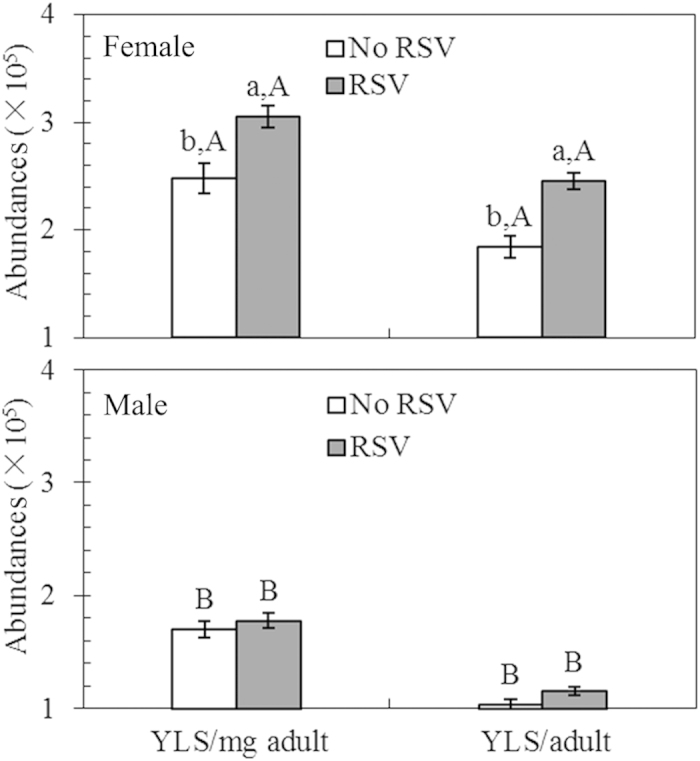
The relative and absolute abundance of yeast-like symbionts (YLS) harbored in female and male adults of *L. striatellus* with (RSV) and without RSV (No RSV) infection. n = 184 and 181 for the infected female and male adults, respectively; n = 156 and 166 for the uninfected female and male adults, respectively. Only significant differences are marked with letters. Different lowercase and uppercase letters show significant differences between the infected and uninfected *L. striatellus* for females or males, and between the females and males for infected or uninfected *L. striatellus* by LSD test at *P* < 0.05.

The relative and absolute abundances of YLS harbored in newly emerged adults of *L. striatellus* were significantly affected by RSV infection (*P* < 0.001 for both relative and absolute abundance), sex (*P* < 0.001 for both relative and absolute abundance) and their interactions (relative abundance: *P* = 0.01; absolute abundance: *P* < 0.001) (see Supplementary Table S2 online). RSV infection significantly increased the abundance of YLS in female adults (mean difference relative abundance: +23.15%; absolute abundance: +33.02%) (both comparisons *P* < 0.05; Fig. 4), but it only marginally increased the abundance of YLS in male adults (mean difference relative abundance: +4.71%; absolute abundance: +10.58%) (both comparisons *P* > 0.05; Fig. 4). Overall, YLS abundances in female adults were significantly higher than in male adults both with RSV (mean difference relative abundance: +71.91%; absolute abundance: +53.06%) and without RSV infection (mean difference relative abundance: +45.88%; absolute abundance: +76.92%) (all comparisons *P* < 0.05; Fig. 4).

### Relationships between RSV and YLS abundance, nymphal stage duration and adult weight of *L. striatellus* with RSV infection

The indirect enzyme linked immunosorbent assay (ELISA) method was used to determine the relative abundance of RSV harbored in female and male adults of *L. striatellus*. The results showed that there were no significant differences in the relative abundance of RSV between females and males (mean OD_405_: 1.619 vs. 1.622; *P* = 0.88; see Supplementary Table S2 and Supplementary Fig. S3 online). Pearson correlation analysis (Table 2) indicated a significant positive correlation between the relative abundance of RSV and YLS in female adults (n = 170, *r* = 0.30, two-tailed, *P* < 0.001), but no significant correlations were found between the relative abundance of RSV and nymphal stage duration (n = 170, |*r|* < 0.10, two-tailed, *P* > 0.24), or between the relative abundance of RSV and adult weight (n = 170, |*r|* < 0.07, two-tailed, *P* > 0.44) for the females and males of *L. striatellus* with RSV infection.

### Effects of RSV infection on the feeding behavior of *L. striatellus*

**Figure 5:**
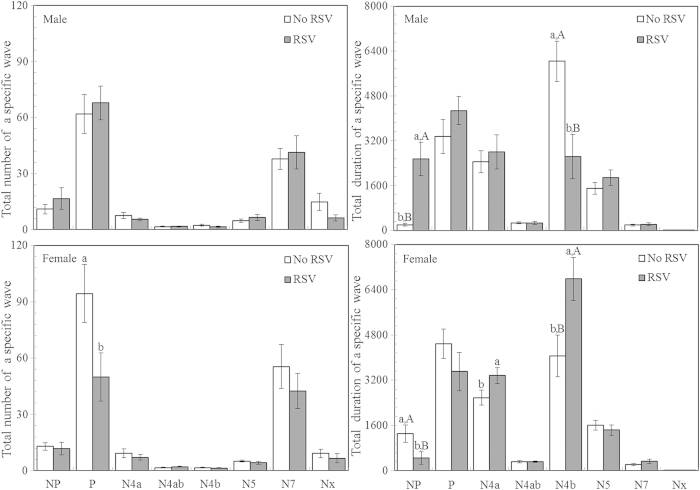
The EPG experiment of feeding behavior for the female and male adults of *L. striatellus* with (RSV) and without RSV (No RSV) infection. NP: Non Penetration waveform; P: Pathway phase, sum of irregular mixed and transition phase prior to N4a; N4a: Sieve element salivation waveform; N4ab: Transition phase between N4a and N4b; N4b: Sieve element ingestion waveform; N5: Xylem feeding waveform; N7: Potential cell penetration; Nx: Unclear waveform. n = 15–20 for each treatment; Only significant differences are marked with letters. Different lowercase and uppercase letters show significant differences between the infected and uninfected *L. striatellus* for females or males, and between the females and males for the infected or uninfected *L. striatellus* by LSD test at *P* < 0.05.

There was a significant interaction effect between RSV infection and sex on the total number of P waveforms (*P* = 0.03), and on the duration of NP (*P* < 0.001), P (*P* = 0.004) and N4b (*P* < 0.001) waveforms. A significant effect of RSV infection (*P* = 0.02) on the duration of N4a waveform was also observed (see Supplementary Table S2 online). The total number of P waveforms was significantly lower for the female adults with RSV infection compared to those without RSV infection (mean difference: -47.08%; *P* < 0.05; Fig. 5). Moreover, RSV infection significantly decreased the duration of NP waveform (mean difference: -71.85%) and significantly increased the duration of N4b waveform (mean difference: +67.20%) for the female adults (both comparisons *P* < 0.05; Fig. 5). The effects of RSV infection on the duration of NP (mean difference: 1176.83%) and N4b (mean difference: -56.44%) waveforms for the males were opposite to the females (both comparisons *P* < 0.05; Fig. 5), showing the existence of sexual dimorphism in the duration of NP and N4b waveforms of *L. striatellus* feeding in response to RSV infection. Significantly increased durations of N4a waveforms were also found for female adults with RSV infection compared to those without RSV infection (mean difference: 32.25%; *P* < 0.05; Fig. 5).

## Discussion

**Figure 6:**
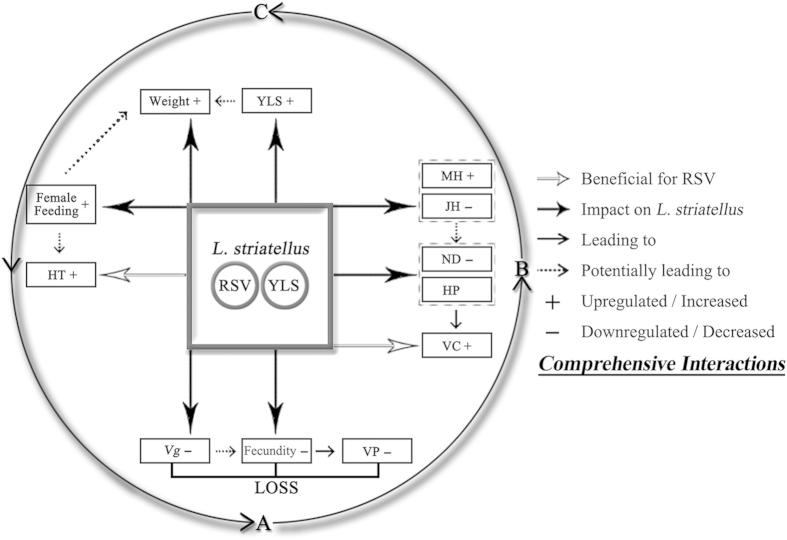
Potential comprehensive interactions between in the RSV-*L. striatellus*-YLS system. Interactions as based the effects of RSV infection on the development, physiology and feeding behavior of its vector *L. striatellus* and on the virus transmission. Generation: A—B: egg stage; B—C: nymph stage; C—A: adult stage; Abbreviation: *Vg*: *vitellogenin* gene; VP: Viruliferous population; VC: vertical circulation; HP: Hatching period; ND: Nymphal stage duration; JH: Juvenile hormone; MH: Molting hormone (Ecdysteroids); YLS: Yeast-like symbiotes; HT: horizontal transmission.

RSV infection of its vector, *L. striatellus,* reduced female fecundity, which would be predicted to negatively impact the spread of the virus across generations and to new hosts. As a way to potentially compensate for this negative effect, RSV infection accelerated nymphal development, increased the weight, enhanced abundance of YLS, and modified the feeding behavior of *L. striatellus*. These virus-mediated changes in vector physiology, endosymbionts and behavior could compensate for reduced fecundity by favoring the transmission of RSV. Based on the known and inferred effects of RSV infection on *L. striatellus*, a conceptual model describing interactions and their potential consequences in the RSV-*L. striatellus*-YLS system is provided in Fig. 6.

Decreases observed in the expression levels of the *Vg* gene in newly emerged female adults along with a decrease in the number of eggs laid per female confirmed previous speculation that RSV infection has deleterious effects on female fecundity^32^,^33^,^34^,^35^. It has been reported that JH regulates *Vg* gene transcription in most hemipterans^10^ and topical application of JH III up-regulated *Vg* gene expression in the brown planthopper, *Nilaparvata lugens*^36^. Given that positive correlations have been found between *JHAMT* expression levels and JH titer^37^, our results are consistent with the same positive relationship linking gene expression levels of *JHAMT* and *Vg.*

JH and the main ecdysteroid, 20-hydroxy-ecdysone (20E), are commonly known for coordinating development and growth of insects^11^. Positive correlation between *CYP307A1* expression and 20E in *Anopheles gambiae* was revealed by Pondeville *et al.* (2013)^38^. It was also reported that 20E blocked innate immunity at the onset of metamorphosis in *Drosophila*^12^, and that JH and ecdysteroids dynamically regulate innate immunity in the fat body during *Bombyx* postembryonic development, with JH acting as an immune-activator and 20E inhibiting innate immunity^13^. The up-regulation of *JHAMT* at 0 h and marginal down-regulation of *CYP307A1* at 24 h, suggested an early immunity activation in RSV-infected *L. striatellus*. However, the dynamic gene expression levels of *JHAMT* and *CYP307A1* that we observed later in *L. striatellus* are consistent with a dominant immunosuppression triggered by RSV infection, with *JHAMT* down-regulated at 60 h and 72 h and *CYP307A1* up-regulated at 0 h, 48 h, 60 h and 72 h. Given that interactions between JH and ecdysteroids play major roles in growth, the immune response characterized by the down-regulation of JH and up-regulation of 20E level should be related to the significantly accelerated nymphal development we observed. Accelerated nymphal development leads to a shorter generation time among RSV-infected individuals. This could favor RSV infection as the shortened generation time might promote RSV spreading by earlier adult migration and dispersal. The shortened generation time could be particularly beneficial in accelerating the vertical cycle of RSV transovarial transmission in the viruliferous population. (i.e., virus vertical circulation). *L. striatellus* is a migratory pest that is able to overwinter in diapause^2^, and the transovarial transmission of RSV may last for as many as 40 generations with a transovarial transmission rate ranging from about 60% to 100% when fed on rice *Oryza sativa* L.^5^. Considering this, the cumulative effects over generations would further magnify this beneficial effect triggered by RSV. Importantly, a shorter juvenile development period is also widely assumed to reduce the risk of predation (or exposure to other mortality factors) prior to reproduction^39^. This could be an additional non-mutually exclusive consequence of RSV infection that further contributes to RSV transmission. Moreover, a recent study showed that knockdown of *Vg* expression due to RNA interference could result in inhibition of the invasion of ovarioles by RSV^8^. Thus from an evolutionary perspective, significantly decreased Vg expression in this study may be an adaptive response to the immunosuppression. In light of the above interactions, RSV-triggered changes in physiology and development to compensate for reduced fecundity in *L. striatellus* could represent the outcome of co-evolutionary interactions between the virus and its vector, since fitness of RSV relies heavily on its vectors’ fitness and ability to move itself to new hosts.

A delay in egg development attributable to RSV infection has been reported in *L. striatellus* by Li *et al.* (2015)^34^, which seems contradictory to our results showing no effect of RSV infection on hatching time, but an effect of accelerated nymphal development. Contrasting effects of RSV infection on *L. striatellus* fecundity have also been reported elsewhere^35^,^40^, and may be due to differences in experimental conditions including temperature, photoperiod, rice cultivar, insect rearing, treatment method, sampling times and ratios of RSV-positive individuals in the progeny from viruliferous mothers^41^. These variable results imply that the effects on development and reproduction triggered by RSV infection on *L. striatellus* might be influenced by environmental or genetic factors. Further studies must be performed to address the differences.

In addition to the beneficial changes in nymphal development associated with RSV infection, we also observed potentially beneficial virus-associated changes in interactions between *L. striatellus* and its primary endosymbionts, YLS. To our knowledge, this is the first such report. It has previously been shown that significantly higher weights were found in the symbiont-harbouring *Nilaparvata lugens* compared with aposymbiotic individuals^42^. Thus the higher weight found in *L. striatellus* may be attributed to the significantly higher abundance of YLS triggered by RSV infection. We found a significant positive correlation between the relative abundance of RSV and the abundance of YLS, and significant increases in both the abundance of YLS and the body weight of adult *L. striatellus* infected with RSV, but only in females. This relationship suggests that RSV and YLS are likely to interact in the process of transovarial transmission in female adults. RSV infection may stimulate YLS propagation, which in turn may supply additional nutritional resources to support insect growth^17^,^18^,^19^. In this way, RSV may indirectly benefit both its vector and itself by increasing the abundance and associated nutritional benefits of YLS^17^,^18^,^19^,^20^,^21^,^22^. This finding represents an initial step in elucidating the interaction between persistent propagative viruses and the primary endosymbionts in their insect vector hosts. These interactions warrant further study due to their implications for the coordinated control of plant diseases and their vectors.

The behavior of insect vectors has profound ecological and evolutionary implications for plant virus transmission^43^. In most cases, the acquisition and inoculation of a plant virus often occurs through the insect vector feeding process. As such, the ability of a plant virus to alter its vectors’ feeding behavior in a manner that facilitates its own transmission would be selectively advantageous^31^. We found that the feeding behavior of *L. striatellus* with and without RSV infection exhibited a sexual dimorphism in the number and duration of NP and N4b waveforms. N4a is the preparation period just prior to N4b, and virtually all studies of feeding behavior of herbivorous insects support the idea that saliva secretion happens prior to ingestion of cells or phloem sap (i.e. N4b in *L. striatellus*)^31^,^44^,^45^,^46^. Thus, our findings of extended duration of N4a (phloem salivation waveforms) in RSV-infected females during the period that is essential for inoculation of persistently transmitted viruses^47^, is directly related to a higher probability of RSV transmission to the host plant. Furthermore, the significant decrease in the number of P waveforms in RSV-infected adult females and the significant decrease in the duration of NP waveform could both save time on non-ingestion probes before the stylet gets to the phloem. Once the stylet reaches the phloem, RSV-infected female *L. striatellus* could sustain ingestion, and thus lead to significantly longer durations of N4a and N4b waveforms compared with the females without RSV infection. As a result, the likelihood of horizontal transmission of RSV infection is changed by alterations in the feeding behavior of infected *L. striatellus* and enhanced RSV transmission would most likely happen in female adults. This scenario is further supported by the agreement between our findings and those of Gingery (1988) in which a difference in RSV transmission efficiency between the females and males was found^7^. A similar case of sexual differences in virus transmission has recently been found in western flower thrips, in which the males spread *Tomato spotted wilt virus* more efficiently than females as a result of feeding behavior modification^31^,^48^.

Taken together, the interactions in the RSV-*L. striatellus*-YLS system we have shown suggest that the RSV-triggered changes in insect development, behavior and endosymbiont abundance could be considered as viral adaptations that counter the potentially negative effects of RSV infection on its insect vector, as have been observed in this and other studies^32^,^33^,^34^,^35^. To our knowledge, this is the first reported evidence of a link between RSV infection of its insect vector, *L. striatellus*, and changes that positively affect the likelihood of viral transmission across generations and between hosts. Our study also suggests that the nature of relationships between a plant virus and its insect vector cannot be simply classified as mutualistic or antagonistic; a more comprehensive and integrated assessment is necessary. The recent whole genome sequencing of the other closely-related rice virus vector, the brown planthopper, *Nilaparvata lugens* (Stål), along with its fungal and bacterial endosymbionts^4^,^49^ provides an excellent rice planthopper model for further investigating plant virus-insect-endosymbionts interactions and the underlying mechanisms.

## Methods

### Small brown planthopper, *Laodelphax striatellus*

Both the high-viruliferous (RSV-carrying rate: ≥90%) and non-viruliferous *L. striatellus* strains originated from an initially half-viruliferous strain (nearly 50% RSV-carrying rate) and were reared for multiple generations on rice seedlings (cv. TN1) separately in glass beakers (diameter: height = 10 cm: 15 cm) as stock populations in a growth incubator (HPG280H; Orient Electronic, Harbin, China) at 26°C with a photoperiod of 14 h/10 h (light/dark). The rice seedlings were fertilized with Kimura-B culture solution, and the glass beakers were enclosed with a piece of nylon mesh after introducing insects onto 2–3 cm high rice seedlings. All rice planthoppers in the colony were provided with fresh seedlings every 10–14 days for sufficient nutrition.

### Hatching period, nymphal stage duration, and adult weight and fecundity

Seventy and 60 male-female pairs of high-viruliferous and non-viruliferous *L. striatellus,* respectively, were reared separately and mated on rice seedlings in glass tube cages (diameter: length = 3 cm: 15 cm) for 2 days. Each mated female was transferred into another glass tube with new rice seedlings to oviposit for one day and then all adults were removed. Glass tubes were checked daily for newly hatched 1st instar nymphs to quantify hatching period (days) of *L. striatellus*. RSV infection was detected by dot-immunoblot assay (DIBA)^50^,^51^ when the 1st instar nymphs grew to 5th instar nymphs. Other newly hatched 1st instar nymphs were transferred into numbered glass tubes (diameter: length = 3 cm: 15 cm) with one nymph per tube, and the nymphs were daily checked for molting. The exuvia was removed and the ecdysis date was recorded to quantify *L. striatellus* nymphal duration. Once the adults emerged, they were identified as females and males, and weighed using precision scales with an accuracy of ± 0.1 μg (Mettler Toledo XP2U). The adults were then transferred into 1.5 ml clear microtubes (Axygen MCT-150-C) for quantification of YLS and RSV as described below. RSV detection was conducted on all adults. The insects chosen for measuring nymphal stage duration and adult weight were used to quantify the YLS and RSV. To test RSV effects on fecundity, 15 male-female pairs of adults with and without RSV infection were reared separately and mated following the same protocol as described above for hatching period. Since female adult *L. striatellus* mates multiple times with male adult, a new male was added into the glass tube when the original male died before the female. One rice seedling per glass tube was planted in Kimura-B culture solution. Glass tubes were enclosed with a piece of nylon mesh, and culture solution was added along the tube wall every 3 days for sufficient nutrition. Cages were checked daily for hatched nymphs and unviable eggs were counted by dissecting rice stems under a stereomicroscope (MOTIC SMZ-168) until the female adults died. RSV detection was conducted by DIBA when the female adult died. The fecundity of *L. striatellus* with and without RSV infection was calculated as the sum of unviable eggs and hatched nymphs.

### RSV detection—Detection of RSV by dot-immunoblot assay (DIBA)

A dot-immunoblot assay (DIBA)^50^,^51^ was conducted to detect RSV infection in *L. striatellus* chosen for measuring the hatching period, nymphal stage duration, adult weight and adult fecundity. Three replicate samples per individual were made and RSV was confirmed by two positives out of three replications.

### RSV detection—Detection of RSV by RT-PCR

Total RNA was isolated following the standard protocol of TRIzol reagent (Invitrogen) from individual newly-molted to 3 day-old (i.e., 0, 24, 48, 60 and 72 h post-ecdysis) 5th instar nymphs and individual newly emerged female adult *L. striatellus* which were used for the quantitative real-time PCR (qRT-PCR) experiment. The concentration and quality of total RNA were determined by a NanoDrop spectrophotometer (Thermo Scientific). The cDNA was synthesized by using the PrimeScrip RT reagent kit (TaKaRa) after treatment with DNAse I (TaKaRa) to remove contaminated genomic DNA. PCR was conducted with 2 × EasyTaq PCR SuperMix (TransGen), using a RSV specific primer pair and RNA specific control primers in duplex PCR as described by Cai *et al.* (2003)^52^. PCR products were analyzed by 1% agarose gels. Amplification fragments were verified by DNA sequencing. Detection of RSV by RT-PCR was conducted before the quantitative real-time PCR (qRT-PCR) analysis of *Vitellogenin*, *CYP307A1* and *JHAMT* as described below.

### Quantification of YLS and RSV in female and male adults of *L. striatellus*

The insects were transferred into 1.5 ml clear microtubes (Axygen MCT-150-C) once the adult *L. striatellus* emerged. They were numbered & identified as females or males, cooled to –22 °C for 80 s and weighed using precision scales with an accuracy of ± 0.1 μg (Mettler Toledo XP2U), and kept in microtubes separately at −80°C in an ultra-cold storage freezer (Thermo Scientific Forma 702, USA) for experiments. To quantify YLS and RSV in the same *L. striatellus*, a modification of indirect ELISA assays was conducted: A 200 μL aliquot of carbonate buffer (50 mM at pH 9.0) was added to each 1.5 ml clear microtube containing *L. striatellus* individuals, and all samples were homogenized in a Tissue Lyser II (Qiagen) by shaking for 1.5 min at 20 Hz with two steel balls. This parameter set can ensure that all tissues and cells can be lysed as fully as possible to release YLS into the buffer. After homogenization, the steel balls were removed with a magnet and washed with 60 μL carbonate buffer to make a final volume of 260 μL in each 1.5 ml clear microtube. YLS in the sample tubes were sedimented by centrifugation at 12000 g for 2 min (5810R centrifuge, Eppendorf). RSV remained in supernatant and was transferred to an ELISA plate with 70 μL supernatant in each well. The supernatant was extracted carefully to prevent the loss of YLS in the sediment), covered with parafilm and incubated overnight at 4°C, three replications were made for each individual. The blocking, washing, and incubation steps were same as the indirect ELISA, except that the color reaction was developed by a conventional alkaline phosphatase-conjugated rabbit anti-mouse IgG (A4312, Sigma Immunochemicals, Labkemi, Stockholm, Sweden) 70 μl/well, diluted 1:20000 in block buffer followed by 70 μl/well pNPP-substrate (Sigma Immunochemicals) diluted in diethanolamine buffer to a final concentration of 1 mg/ml. The reaction was stopped after a 40-min incubation in the dark by the addition of 70 μl/well of 0.1 M EDTA (EDTA: sc-29092), pH 7.5. Negative controls were made using non-viruliferous *L. striatellus*, and positive controls made using RSV purified from infected rice plants. The plates were read with a microtiter plate reader (Model 550; Bio-Rad, Hercules, California, USA) at OD405 to quantify the relative abundance or RSV. The remained 50 μL sediment that contained the YLS was fully homogenized with a vortex to ensure the even distribution, and three replications (10 μL/replication) were made for each individual. Then YLS was quantified by the commonly used hemacytometry according to the method described by Chen *et al.* (2006b)^53^. Excluding the loss, 10 μL homogenate per microtube was used to redetect RSV with DIBA assay for accuracy.

### Quantitative real-time PCR (qRT-PCR) analysis of *Vitellogenin*, *CYP307A1* and *JHAMT*

For qRT-PCR analysis of *vitellogenin* (*Vg*) gene, total RNA was extracted with Trizol (Invitrogen) from newly emerged female adults of the high-viruliferous and non-viruliferous *L. striatellus* strains. For the qRT-PCR analysis of the *CYP307A1* (Genebank ID: KC701468.1) and *JHAMT* (primers provided by Prof. Li, G.Q. from the Department of Entomology, Nanjing Agricultural University) genes, RNA was similarly extracted from newly-molted to 3 day-old (i.e., 0, 24, 48, 60 and 72 h post-ecdysis) 5th instar nymphs of high-viruliferous and non-viruliferous strains of *L. striatellus* (see Supplementary Table S1 for primer sequences). First-strand complementary DNA was synthesized using the PrimeScript™ RT reagent kit (TaKaRa). RSV was detected with RT-PCR as described above, and the qRT-PCR was performed using SYBR^®^ *Premix Ex Taq*™ (Tli RNaseH Plus) (TaKaRa) in combination with a 7500 Real-Time PCR Detection System. Reactions were performed in a 20 μl final volume reaction, using primers in a final concentration of 200 nM and 1 μl of the undiluted *L. striatellus* cDNA template to make the Ct value fall within the suitable range of 15 to 35 based on preliminary experiments. No template was added to negative control reactions. A total of 30 infected and uninfected samples were separately mixed as 1 repeat, and 3 repeats were made for each treatment in the qRT-PCR analysis of the *CYP307A1* and *JHAMT.* A total of 10 infected and uninfected samples were separately mixed as 1 repeat, and 9 repeats were made for each treatment in the qRT-PCR analysis of the *Vg*. ACTIN1 and ARF2 were used as housekeeping genes. The specific primers for all genes used in the qRT-PCR assays are shown in Supplementary Table S1.

### The electrical penetration graph (EPG) recordings

The EPG technique was conducted to monitor feeding behavior of male and female *L. striatellus* adults with and without RSV infection using a GIGA-8 DC EPG amplifier system (EPG system, Wageningen University) as introduced by Tjallingii^54^. Only brachypterous female and male adults were selected from the high-viruliferous and non-viruliferous strains of *L. striatellus. L. striatellus* individuals were carefully connected to a gold wire (diameter: length = 18.5 μm: 3 cm) with conductive silver glue on their dorsum. After 1 h starvation, *L. striatellus* individuals were linked to the amplifier. To complete the electronic circuit, they were connected to the same position of stem area of each rice plant above the soil at 2nd or 3rd internode. The experiment was conducted in a greenhouse at 26.5 ± 1°C with 70 ± 10% humidity at a photoperiod of 14 h/10 h (light/dark). In order to reduce technical error, recordings were made on 4 channels simultaneously. Probing behavior was recorded for 6 h continuously and 4 h of records starting from the beginning of feeding were analyzed using EPG Stylet + a software (Wageningen Agricultural University, 2012). At least 15 replicates (15–20) per treatment were conducted. All recorded signals were analyzed.

Supplementary Fig. S1 shows typical DC-EPG waveform patterns produced by *L. striatellus* feeding on rice seedlings, based on the analyses in relation to other planthopper studies^46^,^55^,^56^,^57^. In this analysis, non-penetration (NP) waveform correlated with the absence of feeding. Pathway phase (P) indicated that the stylet of *L. striatellus* was inserted into the plant; it was irregular with increased amplitude as reported by Seo *et al.* (2009) in BPH^46^. The N5 waveform occurred occasionally during the pathway period and it exhibited a consistent shape close to that found by Seo *et al.* (2009), which is identified as xylem feeding waveform^46^. N4a has been suggested as sieve element salivation waveform, and N4b has been confidently attributed to sieve element feeding phase^46^,^56^,^57^,^58^. N4ab was found as a transition phase between the waveforms of N4a and N4b, and a similar phase was also found by Seo *et al.* (2009)^46^. The N7 waveform was classified as potential drops since it suddenly dropped from active pathway activities described by Ghaffar *et al.* (2011)^56^, and it was correlated with cell penetration of aphids described by Tjallingii (1988)^58^. The Nx waveform occasionally occurring during the period of N4b waveform (Fig. 1) was first found in our study.

### Data analysis

Statistical analysis of all data was performed using SPSS 20.0. Levene’s test was used to test the homoscedasticity of variances (*P* > 0.10) and Shapiro–Wilk test was analyzed for normality (*P* > 0.05). Data were transformed if necessary to meet normality assumptions. The developmental durations of the 1st–5th instar nymphs, adult body weights, YLS abundance and EPG waveforms (square-root transformation for total number and natural log transformation for total duration of specific waveforms to improve model fit) of the female and male adults were analyzed separately using two-way analysis of variance (ANOVA), with RSV as main factor (infected vs. uninfected) and sex (female vs. male) as sub-factor. Since the 1st–5th instar nymphal stage durations of *L. striatellus* were measured on the same individuals over time, nymphal durations were also analyzed by two-way repeated measures ANOVA with RSV as main factor (infected vs. uninfected) and sex (female vs. male) as sub-factor, and nymphal instars as repeat effects. Two-way ANOVAs were also used to analyze the effects of RSV infection on the gene expression levels of *CYP307A1* and *JHAMT* for the 5th instar nymphs across sampling times from newly molted to the 3 days post-molt. One-way ANOVAs were also used to analyze the effects of RSV (infected vs. uninfected) on the developmental duration of eggs (hatching period), the number of eggs laid per female and the gene expression level of *Vg* in female adults of *L. striatellus*. If significant effects of RSV infection, sex (sampling time) or their interactions on the above variables were found, the least significant difference (LSD) test was further used to compare the means between the infected and uninfected *L. striatellus* or between females and males of *L. striatellus* at *P* < 0.05. For the *L. striatellus* with RSV infection, Pearson correlation analysis was conducted to investigate the relationships between the relative abundance of RSV with YLS abundance, adult weight and nymphal stage duration for both females and males of *L. striatellus*. Absolute abundance (YLS/adult) rather than relative abundance (YLS/mg adult) of YLS was chosen for representative of the YLS abundance to avoid the influence from the parameter of *L. striatellus* adult weight in the Pearson correlation analysis. Necessary test was conducted before the Pearson correlation analysis to rule out the self-correlation (Durbin-Watson) and collinearity (Collinearity diagnosis).

## Additional Information

**How to cite this article**: Wan, G. *et al.* Rice stripe virus counters reduced fecundity in its insect vector by modifying insect physiology, primary endosymbionts and feeding behavior. *Sci. Rep.* **5**, 12527; doi: 10.1038/srep12527 (2015).

## Supplementary Material

Supplementary Information

## Figures and Tables

**Table 1:** Total nymphal stage duration and adult weight of the females and males of *L. striatellus* with and without RSV infection (means ± SE).

**Sex**	**RSV infection**	**Nymphal stage duration (days)**	**Adult weight (mg)**
Female	No RSV	17.65 ± 0.30 a,A	0.763 ± 0.020 b,A
	RSV	16.47 ± 0.21 b,A	0.815 ± 0.012 a,A
Male	No RSV	16.59 ± 0.26 a,B	0.677 ± 0.011 a,B
	RSV	15.48 ± 0.15 b,B	0.689 ± 0.007 a,B

n = 184 and 181 for the infected females and males, respectively; n = 156 and 166 for the uninfected females and males, respectively. No RSV—no infection of RSV; RSV—infection of RSV. Different lowercase letters show significant differences between the infected and uninfected *L. striatellus* for females or males, and different uppercase letters show significant differences between the females and males for infected or uninfected *L. striatellus* by LSD test at *P* < 0.05, respectively.

**Table 2:** Correlations between the relative abundance of RSV (OD_405_) and the abundance of YLS (×10^5^/adult), nymphal stage duration (days) and adult weight (mg) of *L. striatellus* by the Pearson correlation analysis (*r*(*P*) values).

**Sex**	**YLS/adult**	**Nymphal stage duration**	**Adult weight**
Female	0.30 (*P* < 0.001)	-0.10 (0.24)	0.07 (0.44)
Male	0.05 (0.56)	-0.05 (0.54)	0.01 (0.73)

n = 170 for females and males respectively.
