# Supplementary material for: Rice stripe virus counters reduced fecundity in its insect vector by modifying insect physiology, primary endosymbionts and feeding behavior
Source: Sci Rep. 2015 Jul 27;5:12527. doi: 10.1038/srep12527 (PMC4648468; doi:10.1038/srep12527)
Supplement: Supplementary Information [file srep12527-s1.pdf]

Supplementary Information

Rice stripe virus counters reduced fecundity in its insect vector by modifying insect physiology, primary endosymbionts and feeding behavior

Guijun Wan[1], Shoulin Jiang[1], Wenjing Wang[1], Guoqing Li[1], Xiaorong Tao[2,*], Weidong Pan[3], Gregory A. Sword[4] & Fajun Chen[1,*]

[1]Department of Entomology, College of Plant Protection, Nanjing Agricultural University, Nanjing 210095, China.

[2]Department of Plant Pathology, College of Plant Protection, Nanjing Agricultural University, Nanjing 210095, China.

[3]Beijing Key Laboratory of Bioelectromagetics, Institute of Electrical Engineering, Chinese Academy of Sciences, Beijing 100190, China.

[4]Department of Entomology, Texas A&M University, College Station, TX 77843, USA.

[*]Correspondence to F.J.C. (fajunchen@njau.edu.cn) or X.R.T. (taoxiaorong@njau.edu.cn)

Supplementary Tables

Supplementary Table S1 | Primers used to measure the relative transcript levels of *vitellogenin* (*Vg*), *CYP307A1* and *JHAMT* of small brown planthopper, *Laodelphax striatellus* in the qRT-PCR experiment

Primer	Sequence (5' to 3')	GeneBank	Description
ACTIN1-F	GTATCATCACCAACTGGGACGAC	KC683802.1	housekeeper gene
ACTIN1-R	GTCATCTTCTCACGGTTGGC		
ARF2-F	GCCCTATCTGCCGAAGAAATAAGAG	JF728807.1	housekeeper gene
ARF2-R	ACGAGCAGCAATGTCATCAATAAG		
Vg-F	CAACATTCTGCCCCAATCCG	KC469580.1	*Vitellogenin*
Vg-R	TTGGCAGCTCATCAACATCGT		
CYP307A1-F	CTCGGCAAACATCTGAAACGG	KC701468.1	cytochrome P450, family 307, subfamily A
CYP307A1-R	TCAACATAGTCGCCAGACGAAG		
JHAMT-F	CCCCTTGTTCCAAATGATGCC	From Li GQ	JH acid methyltransferase
JHAMT-R	TGTCTGATATTTTCTGAGCCGAAG		

Supplementary Table S2 | Combined *L. striatellus* analysis of variance (ANOVA) results table. One-way ANOVA results are presented for the effects of rice stripe virus (RSV) infection on (i) hatching period and (ii) no. of eggs per female, and (iii) for the effect of Sex on the relative abundance of RSV. Two-way ANOVA results are presented for the effects of RSV infection, Sex and their interactions on (iv) nymphal stage duration, (v) adult weights, (vi) abundance of yeast-like symbionts (YLS), and (vii) the no. and duration of EPG (electrical penetration graph) waveforms.

Parameter	RSV [a]			Sex [b]			RSV × Sex		
	MS [c]	df	F (*P*)	MS	df	F (*P*)	MS	df	F (*P*)
Hatching period	14.910	1,3232	0.90 (0.34)						
Nymphal stage duration									
1st instar	1.912	1,683	10.22 (0.001)	0.148	1,683	0.79 (0.38)	0.206	1,683	1.10 (0.30)
2nd instar	2.775	1,683	2.09 (0.15)	22.396	1,683	16.87 (<0.001)	0.678	1,683	0.51 (0.48)
3rd instar	3.547	1,683	4.93 (0.03)	2.864	1,683	3.98 (0.047)	0.813	1,683	1.13 (0.29)
4th instar	6.405	1,683	6.71 (0.01)	3.102	1,683	3.25 (0.07)	0.134	1,683	0.14 (0.71)
5th instar	32.156	1,683	8.12 (0.005)	18.850	1,683	4.76 (0.03)	2.417	1,683	0.61 (0.44)
Total	204.132	1,683	27.99 (<0.001)	168.542	1,683	23.11 (<0.001)	0.511	1,683	0.07 (0.80)
Adult									
Body weight (mg)	0.326	1,683	10.28 (0.001)	4.747	1,683	149.87 (<0.001)	0.008	1,683	0.24 (0.63)

No. of eggs / female	4083.333	1,28	8.58 (0.007)						
YLS abundance (×10^5)									
Individuals / mg adult (Relative)	16.871	1,683	11.12 (<0.001)	170.936	1,683	112.67 (<0.001)	10.185	1,683	6.77 (0.01)
Individuals / adult (Absolute)	20.466	1,683	26.30 (<0.001)	171.765	1,683	220.73 (<0.001)	9.198	1,683	12.01 (<0.001)
RSV abundance				0.001	1,338	0.03 (0.88)			
No. of EPG waveforms [d]									
NP	87.612	1,62	0.80 (0.38)	31.759	1,62	0.29 (0.60)	186.236	1,62	1.72 (0.20)
P	5185.546	1,62	2.24 (0.14)	1250.087	1,62	0.54 (0.47)	10903.982	1,62	5.01 (0.03)
N4a	71.208	1,62	1.33 (0.25)	47.650	1,62	0.89 (0.35)	0.005	1,62	0.001 (0.98)
N4ab	1.337	1,62	1.13 (0.29)	1.147	1,62	0.97 (0.33)	1.031	1,62	0.87 (0.36)
N4b	1.693	1,62	0.73 (0.40)	1.925	1,62	0.83 (0.37)	2.684	1,62	1.16 (0.29)
N5	2.199	1,62	0.21 (0.65)	16.332	1,62	1.56 (0.22)	27.818	1,62	2.73 (0.10)
N7	383.985	1,62	0.32 (0.57)	1439.943	1,62	1.20 (0.28)	1152.686	1,62	0.96 (0.33)
Nx	476.079	1,62	2.21 (0.14)	103.402	1,62	0.48 (0.50)	134.371	1,62	0.62 (0.44)
Duration of EPG waveforms [d]									
NP	10495244.868	1,62	2.56 (0.12)	6477533.942	1,62	1.58 (0.21)	46687232.824	1,62	13.68 (<0.001)
P	29744.827	1,62	0.02 (0.89)	401555.168	1,62	0.27 (0.61)	12289748.968	1,62	9.36 (0.004)
N4a	5563562.193	1,62	5.41 (0.02)	781572.508	1,62	0.76 (0.39)	1768615.475	1,62	1.74 (0.20)

N4ab	2.905	1,62	3.62 (0.06)	0.008	1,62	0.01 (0.93)	2.643	1,62	3.42 (0.07)
N4b	1800643.565	1,62	1.27 (0.27)	453705.465	1,62	0.32 (0.58)	20097734.277	1,62	18.00 (<0.001)
N5	183305.091	1,62	0.69 (0.41)	5313.191	1,62	0.02 (0.88)	83266.427	1,62	0.31 (0.58)
N7	87533.388	1,62	0.11 (0.75)	7957.581	1,62	0.01 (0.94)	48468.668	1,62	0.06 (0.80)
Nx	317.422	1,62	0.93 (0.34)	10.239	1,62	0.03 (0.87)	652.414	1,62	1.94 (0.17)

[a] RSV - RSV infection vs. non-RSV infection;

[b] Sex - Female vs. male;

[c] MS - Mean square;

[d] Square-root transformation to improve model fit.

Supplementary Table S3 | Two-way analyses of variance (ANOVAs) of the effects of rice stripe virus (RSV) infection, sampling time, and their interactions on gene expression levels of *CYP307A1* and *JHAMT* for 5th instar nymphs; and one-way ANOVA of RSV infection on gene expression level of *Vg* for female adults of *L. striatellus.*

Genes	RSV [a]			Sampling time [b]			RSV × Sampling time		
	MS [c]	df	F (*P*)	MS	df	F (*P*)	MS	df	F (*P*)
CYP307A1	4.338	1,20	36.10 (<0.001)	5.927	4,20	49.32 (<0.001)	0.663	4,20	5.52 (0.004)
JHAMT	0.906	1,20	6.66 (0.02)	2.372	4,20	17.45 (<0.001)	1.305	4,20	9.60 (<0.001)
Vg	0.291	1,16	4.89 (0.04)						

[a] RSV - RSV infection vs. non-RSV infection;

[b] Sampling time - 0h, 24h, 48h, 60h and 72h after the molting time of the 5th instar nymphs;

[c] MS - Mean square.

Supplementary Figures

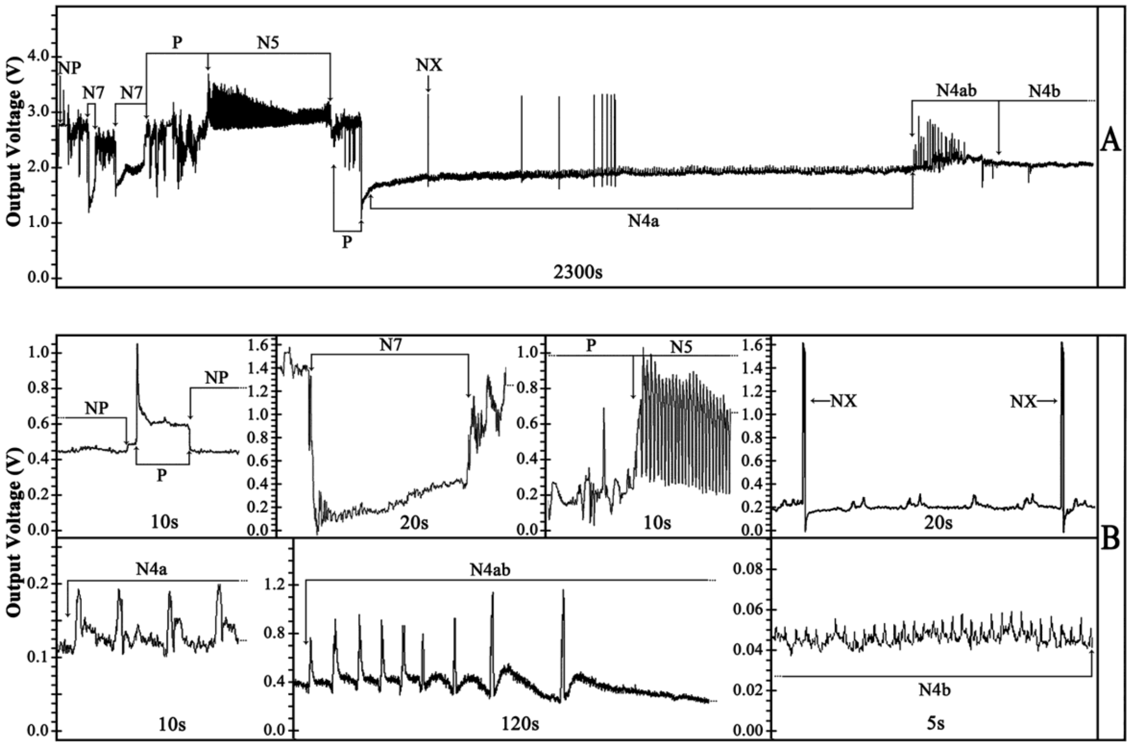

Supplementary Figure S1 | Classification of electrical penetration graph (EPG) waveforms recorded for the small brown planthopper, *L. striatellus,* feeding on rice plants. A: Overall typical waveforms in 2300s; B: Transitional phases and specific waveforms, that is, NP: Non Penetration waveform; P: Pathway phase, sum of irregular mixed and transition phase prior to N4a; N4a: Sieve element salivation waveform; N4ab: Transition phase between N4a and N4b; N4b: Sieve element ingestion waveform; N5: Xylem feeding waveform; N7: Potential cell penetration; Nx: Unclear waveform.

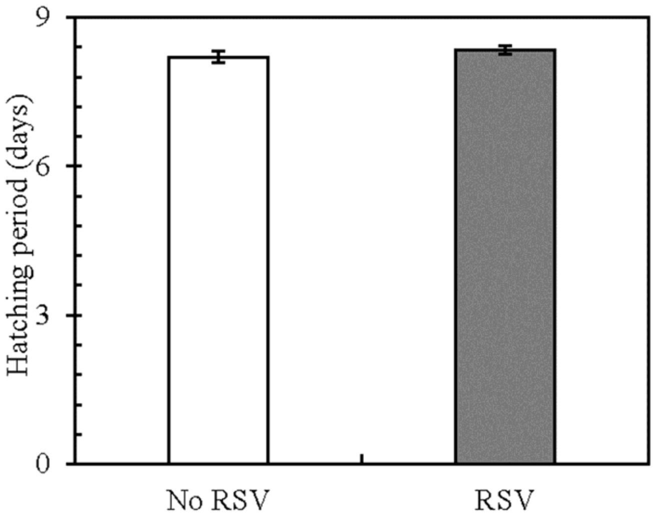

Supplementary Figure S2 | The hatching period of small brown planthopper, *L. striatellus* infected and uninfected with *Rice stripe virus* (RSV). No RSV - *L. striatellus* eggs uninfected by RSV; RSV - *L. striatellus* eggs infected by RSV; n=1,655 for the infected *L. striatellus* eggs and n=1,579 for the uninfected *L. striatellus* eggs; There is no significant difference between the individuals with RSV infection and those without RSV infection by LSD test at $P<0.05$.

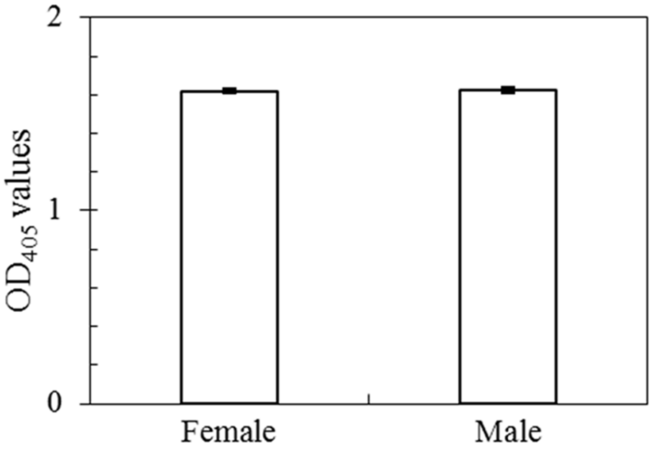

Supplementary Figure S3 | The relative abundance of RSV harbored in female and male adults of *L. striatellus* with RSV infection. n=170 for female and male adults of *L. striatellus* with RSV infection, respectively; There was no significant difference between the female and male adults by LSD test at $P<0.05$.
